# Lifetime antipsychotic medication and cognitive performance in schizophrenia at age 43 years in a general population birth cohort

**DOI:** 10.1016/j.psychres.2016.10.085

**Published:** 2017-01

**Authors:** Anja P. Husa, Jani Moilanen, Graham K. Murray, Riikka Marttila, Marianne Haapea, Irina Rannikko, Jennifer H. Barnett, Peter B. Jones, Matti Isohanni, Anne M. Remes, Hannu Koponen, Jouko Miettunen, Erika Jääskeläinen

**Affiliations:** a Department of Psychiatry, Research Unit of Clinical Neuroscience, University of Oulu, P.O. Box 5000, 90014 Oulu, Finland; b Medical Research Center Oulu, Oulu University Hospital and University of Oulu, Oulu, Finland; c Department of Psychiatry, Oulu University Hospital, P.O. Box 26, 90029 OYS, Oulu, Finland; d Department of Psychiatry, University of Cambridge, Box 189 Addenbrooke's Hospital, Cambridge CB2 0QQ, United Kingdom; e Behavioural and Clinical Neuroscience Institute, University of Cambridge, Herchel Smith Building, Forvie Site, Cambridge Biomedical Campus, Cambridge CB2 0SZ, United Kingdom; f Center for Life Course Health Research, University of Oulu, P.O. Box 5000, 90014 Oulu, Finland; g Department of Diagnostic Radiology, Oulu University Hospital, P.O. Box 50, 90029 OYS, Finland; h Cambridge Cognition Ltd., Tunbridge Court, Bottisham, Cambridge, United Kingdom; i Department of Psychiatry, University of Cambridge, Herchel Smith Building, Cambridge CB2 0SZ, United Kingdom; j Institute of Clinical Medicine – Neurology, University of Eastern Finland, P.O. Box 1627, 70211 Kuopio, Finland; k Department of Neurology, Kuopio University Hospital, Kuopio, Finland; l University of Helsinki and Helsinki University Hospital – Psychiatry, P.O. Box 22, 00014 Helsinki, Finland; m Oulu Occupational Health, Hallituskatu 36 B, 90100 Oulu, Finland

**Keywords:** Psychosis, Cognition, Treatment, Cross-sectional, Adverse effect

## Abstract

This naturalistic study analysed the association between cumulative lifetime antipsychotic dose and cognition in schizophrenia after an average of 16.5 years of illness. Sixty participants with schizophrenia and 191 controls from the Northern Finland Birth Cohort 1966 were assessed at age 43 years with a neurocognitive test battery. Cumulative lifetime antipsychotic dose-years were collected from medical records and interviews. The association between antipsychotic dose-years and a cognitive composite score based on principal component analysis was analysed using linear regression. Higher lifetime antipsychotic dose-years were significantly associated with poorer cognitive composite score, when adjusted for gender, onset age and lifetime hospital treatment days. The effects of typical and atypical antipsychotics did not differ. This is the first report of an association between cumulative lifetime antipsychotic dose and global cognition in midlife schizophrenia. Based on these data, higher lifetime antipsychotic dose-years may be associated with poorer cognitive performance at age 43 years. Potential biases related to the naturalistic design may partly explain the results; nonetheless, it is possible that large antipsychotic doses harm cognition in schizophrenia in the long-term.

## Highlights

• Higher antipsychotic exposure related to poorer cognition in midlife schizophrenia.
• Potential biases related to this naturalistic design may contribute to the effects.
• Higher antipsychotic doses may negatively affect cognitive course in schizophrenia.
• Preventing or attenuating cognitive recovery may be an explanation for the results.

## 1 Introduction

Neurocognitive deficits occur in the majority of persons with schizophrenia (Heinrichs and Zakzanis, 1998, Keefe et al., 2005). They are present before the first psychotic episode, remain relatively stable over the illness course (Bora and Murray, 2014, Zipursky et al., 2013) and are strongly associated with functional outcome (Rajji et al., 2014).

Antipsychotic medication is the foundation of treatment recommendations in schizophrenia, yet the associations of antipsychotic medication with cognition, especially in the long-term, after 5 or more years of illness, remain largely unclear (Husa et al., 2014). The cognitive effects of antipsychotic medication have mostly been studied early in the course of illness in relatively short follow-ups ranging from 1 to 3 weeks to 2–3 years. Meta-analyses of these studies have found mild to moderate cognitive improvements associated with the use of both atypical (Désaméricq et al., 2014, Woodward et al., 2005) and typical (Mishara and Goldberg, 2004) antipsychotic medication in schizophrenia.

However, naturalistic, cross-sectional studies have suggested that higher doses of antipsychotics (Élie et al., 2010, Hori et al., 2006, Torniainen et al., 2012) or antipsychotic polypharmacy (Hori et al., 2006) may be associated with poorer cognitive functioning in schizophrenia, supported also by the finding of a positive effect of dose-reduction on cognition (Kawai et al., 2006, Takeuchi et al., 2013).

Very little is known about the effects of antipsychotic medication in the long-term (Leucht et al., 2012). In particular, the effects of several years or lifetime treatment with antipsychotics on global cognition in schizophrenia have not yet been studied. Because many schizophrenia patients receive antipsychotic treatment for several years or permanently, it is imperative to study the effects of not only short-term but also lifelong antipsychotic treatment. Randomised controlled trials (RCTs) are able to primarily determine the efficacy and adverse effects of a treatment, but they do not allow a more detailed and long-term assessment of adverse effects (Young et al., 2015). Naturalistic samples, however, offer an optimal setting for investigating the long-term effects of medication (Wang et al., 2011), that often are impossible to study in RCTs. In the Northern Finland Birth Cohort 1966 (NFBC 1966), higher lifetime cumulative doses of antipsychotic medication were associated with poorer performance at age 34 and a decline in verbal learning and memory between ages 34 and 43 years in schizophrenia (Husa et al., 2014). We wanted to continue this research line to investigate the effects of lifetime cumulative dose of antipsychotic medication on a more comprehensive measure of cognition in midlife in a larger, partly overlapping sample.

This study aimed to analyse the association between cumulative lifetime antipsychotic dose and cross-sectional global cognition in schizophrenia at the age of 43 years. Our hypothesis was that higher lifetime antipsychotic dose would be associated with poorer cognition, even when potential confounders, such as severity and duration of illness, are taken into account.

## 2 Methods

### 2.1 Sample

#### 2.1.1 Participants

The participants of this study were members of the Northern Finland Birth Cohort 1966. The NFBC 1966 is an unselected, general population birth cohort identified during mid-pregnancy based on an expected delivery date during 1966 in the provinces of Lapland and Oulu. It comprises 12 058 live-born children, representing 96% of all births in the region (Rantakallio, 1969). Permission to gather data was obtained from the Ministry of Social and Health Affairs. The study design was approved by the Ethical Committee of the Northern Ostrobothnia Hospital District. The study was carried out in accordance with The Code of Ethics of the World Medical Association (Declaration of Helsinki) for experiments involving humans.

#### 2.1.2 Case identification

The NFBC 1966 members with a lifetime psychosis diagnosis were identified using data from national registers. Psychosis diagnoses by the end of 1997 were detected from the Care Register for Health Care (formerly Finnish Hospital Discharge Register) and these diagnoses were validated using hospital notes (Isohanni et al., 1997, Moilanen et al., 2003). In addition, newer psychosis cases were detected based on Care Register for Health Care on those registered first time for a psychosis between 1998 and 2008; Social Insurance Institution of Finland register data on sick leaves or disability pensions due to psychosis, or the right to reimbursement for psychoactive medication due to psychosis by the end of 2008; or having reported a psychosis or current antipsychotic use (at least 300 mg chlorpromazine equivalent) in 1997 in a questionnaire data collection (Haapea et al., 2010).

Based on this procedure, 257 NFBC 1966 members with a psychosis diagnosis and known address were invited and 99 (38.5%) individuals participated in a psychiatric interview and examination in 2008–2011 at an average age of 43 years. The examination included the SCID I interview (First et al., 2002) leading to DSM-IV lifetime diagnosis. Based on diagnostic interview and information from national registers, 69 individuals had a diagnosis of schizophrenia spectrum disorder. In the end, 60 (87.0%) were able to complete the cognitive test battery and had adequate information on antipsychotic medication. Of these 50 (83%) had a DSM-IV lifetime diagnosis of schizophrenia, 6 (10%) schizoaffective, 2 (3%) schizophreniform and 2 (3%) delusional disorder. Hereafter, the term schizophrenia is used for schizophrenia and other schizophrenia spectrum disorders. Part of this sample (40 cases, 67%) went through the same diagnostic interviews as well at 34 years of age when participating in another psychiatric examination where we analysed associations between lifetime antipsychotic medication and change of verbal learning and memory in schizophrenia between ages 34 and 43 years (Husa et al., 2014). The mean duration of illness of the sample was 16.5 years (SD 6.0) and average age was 43.1 years (SD 0.8).

In addition, 450 non-psychotic NFBC 1966 members from all around Finland were invited to participate in the same psychiatric interviews and cognitive assessment in 2008–2011. 191 (42.4%) control subjects with an average age of 43.8 years (SD 0.8) and available cognitive test results were included in the final analyses.

Written informed consent was obtained from all cases and controls. Participating cases did not differ from non-participating cases in gender, number of cumulative lifetime hospital treatment days or occupational status. Compared to non-participants, participating cases had significantly lower education (basic education 28% vs. 15%, secondary education 62% vs. 85%, tertiary education 10% vs. 0%) (p=0.001), lower age of illness onset (mean 30.1 years vs. 26.6 years) (p=0.002), they were more often on a disability pension (26% vs. 50%) (p=0.001), and had more often diagnosis of narrow schizophrenia (68% vs. 84%) (p=0.024). Participating controls did not differ from all non-psychotic members of the NFBC 1966 in gender, education or disability pension status. Compared to non-participants, participating controls were more often working (71% vs. 95%) (p<0.001).

### 2.2 Data on antipsychotic medication

Information on the lifetime use of antipsychotic medication, until the day the person was examined in the 43-year study, was gathered in 2007–2014 by a careful review of hospital, outpatient and health centre medical records of all cases from everywhere in Finland. This information was used to calculate the cumulative lifetime antipsychotic dose, expressed as dose-years of a daily dose of 100 mg chlorpromazine equivalent. Medication data was not only based on prescribed medication but if there was indication that a patient had not taken medication it was taken into account in the estimation of lifetime doses. This procedure is described in more detail in our previous work (Husa et al., 2014). Additionally, current and earlier use of antipsychotic and other psychiatric medication was ascertained in an interview in the 43-year study.

### 2.3 Neuropsychological assessment and cognitive composite score

The neurocognitive performance of all cases and controls was evaluated using a cognitive test battery comprising the Abstraction Inhibition and Working Memory task (AIM; Glahn et al., 2000), California Verbal Learning Test (CVLT; Delis et al., 1987), Visual Object Learning Test (VOLT; Glahn et al., 1997), Verbal fluency (Lezak et al., 2004), Visual series (WMS-III; Wechsler, 2008), Vocabulary (WAIS-III; Wechsler, 2005), Digit Span (WAIS-III) and Matrix reasoning (WAIS-III). The cognitive tests were chosen for the versatile assessment of different aspects of cognitive functioning, including executive function, working memory, verbal and visual learning, vocabulary and visual reasoning. From these tests, the following variables were obtained and used in analyses: total score of the first five learning trials of CVLT and total scores of all other cognitive tests. Global cognitive performance (cognitive composite score) was based on a principal component analysis (PCA) of these eight cognitive test variables (see Section 2.5).

### 2.4 Background variables and covariates

The analyses were adjusted for gender, age of illness onset, markers of severity of illness (logarithmic transformation of cumulative lifetime number of psychiatric hospital treatment days until the study, remission, PANSS positive, negative and disorganisation symptoms), educational level and benzodiazepine use at the time of the study.

Age of illness onset was verified from medical records and defined as the age when the subject had first evident psychotic symptoms.

Level of basic education (O level, 9 years or A level, 12 years) and vocational education (none, course or school, currently studying, college, polytechnic, or university) was obtained in a questionnaire in the 43-year study. These were combined as the level of education: Basic=O level with low vocational education (none, course or school, or currently studying), secondary=O level with high vocational education (college, polytechnic, or university), or A level with low vocational education, and tertiary=A level with high vocational education.

The occupational status of the subjects at the time of the 43-year study was classified into two categories: all subjects were classified as (1) working if they were studying, on maternity leave or in full-time or part-time work and (2) not working if they were unemployed, retired or outside of working life for other reasons. Information was gained in the interview at the 43-year study and for those with missing information it was ascertained from Finnish Centre for Pensions registers.

School marks at 16 years of age were based on information acquired from registers of the national application system for secondary education after compulsory schooling (Isohanni et al., 1998). The Finnish school marks range from 4 to 10, where 4 is defined rejected, 5–6 poor, 7–8 satisfactory and 9–10 excellent. The mean scores of all subjects were calculated from school reports. The school subjects included the following theoretical subjects: native language, reading; native language, literal; second, third, fourth and fifth language; mathematics; chemistry; physics; history; biology; geography; religion and civics. Also the following practical subjects were included: physical education, music, drawing, craft, domestic science, commercial subjects, typewriting and agriculture.

Alcohol abuse diagnosis includes subjects with an earlier or current diagnosis of both alcohol abuse and dependency. The information was ascertained in the SCID I interview at the 43-year study.

PANSS (Positive and Negative Syndrome Scale; Kay et al. (2000)) scores were obtained in a PANSS specific interview at the 43-year study. PANSS total symptoms were used to measure psychopathological symptoms from the period of 1 week before the interview and they were divided into symptomatic categories of positive, negative and disorganisation symptoms based on the model described by van der Gaag et al. (2006). Additionally, PANSS was used to determine remission status (described below).

The Severity of Illness subscale of the CGI (Clinical Global Impression; Guy, 2000) ranges from 1 (not ill at all) to 7 (among the most extremely ill), and was included in the interview at the 43-year study.

Information on the cumulative number of hospital treatment days of the cases was derived from the Care Register for Health Care (formerly Finnish Hospital Discharge Register).

Drug Attitude Inventory (DAI-10; Awad, 1993) is a questionnaire which assesses attitude towards antipsychotic medication that was a part of the 43-year study. DAI-10 includes 10 dichotomic (yes/no) statements that were the most sensitive in separating between good and poor adherence in a longer 30-question version (DAI-30; Hogan et al., 1983) that was designed to predict adherence to antipsychotic treatment. In DAI-10 the total score ranges between −10 and +10 with a higher value reflecting better adherence.

Psychiatric treatment status was based on information from interview at the 43-year study, where the participants were asked about previous and current psychiatric treatment contacts (place and time of starting the contact, frequency of visits). This information was classified into four categories: no treatment contact, non-regular outpatient treatment (less frequent than once per month or of unknown frequency), regular outpatient treatment (visits in the mental health office or in a few cases outpatient rehabilitation group at least once and mostly 1–4 times per month), and inpatient/institution (if the case was in psychiatric hospital treatment or lived in a sheltered home).

Remission was determined by the Andreasen symptomatic criteria (Andreasen et al., 2005), the symptoms were required not to be present at the time of the assessment and additionally the subject had to not have been in psychiatric hospital treatment for 6 months prior to the study in order to be classified as being in remission.

### 2.5 Statistical analyses

Mean cognitive test scores and cognitive composite scores of cases and controls were compared using independent samples *t*-tests.

The measure of global cognitive performance was based on a principal component analysis (PCA) of cognitive test variables (Immediate free recall of trials 1–5 of CVLT, total scores of VOLT, AIM, Verbal fluency, Visual series, Vocabulary, Digit Span and Matrix reasoning) performed separately for cases and controls. Missing values of cognitive tests were imputed by multiple imputing (20 different datasets) (Rubin, 1987) where missing values were predicted based on non-missing cognitive test values. The method of imputation was MCMC and model type for scale cognition scores was linear regression. Two cases and 3 controls did not have VOLT score, 1 case did not have AIM score, 1 control did not have CVLT or VOLT scores, 3 cases and 2 controls did not have VOLT or AIM scores, 1 control was missing all other but CVLT, VOLT and AIM scores. The PCA resulted in one cognitive factor (cognitive composite score) for cases and two factors for controls, which were forced into one factor to enable comparison with cases. In the PCA eigenvalue was set as >1. In cases, total variance was explained 51.9% by one factor. Communalities of cognitive tests ranged between 0.34 and 0.65 and factor loadings between 0.51 and 0.78.

The association between the cognitive composite score and antipsychotic dose-years was analysed using linear regression analysis only in cases with schizophrenia. Dose-years of any antipsychotics were used as a continuous variable in the primary analyses and dose-years of typical and atypical antipsychotics separately in secondary analyses. The natural logarithm of antipsychotic dose-years was used as the predictor variable. The effects of antipsychotic dose-years in the linear regression models are presented with unstandardised regression coefficients (B) and their standard error (SE), standardised regression coefficients (Beta) and statistical significance values. Furthermore, using the model with only gender and age of illness onset as covariates, we illustrate the effect of antipsychotic dose with adjusted R^2^ statistics. As sensitivity analyses, all regression analyses were performed with inverse probability weighting for the variables in which the cases had selective attrition (age of illness onset, educational level, occupational status and diagnosis). Spearman's correlation was used to analyse the association between the cognitive composite score, lifetime dose-years of any antipsychotics and background variables. P-values<0.05 were considered statistically significant. The analyses were performed with IBM SPSS Statistics 21 (IBM Corp., 2012).

## 3 Results

### 3.1 The characteristics of the sample

The characteristics of the sample are described in Table 1. The sample consisted of 60 cases (33 males, 55%) with lifetime schizophrenia and 191 non-psychotic controls (94 males, 49%).

### 3.2 The characteristics of medication use

At the time of the study antipsychotic medication was used by 51 (85%) cases and one (1%) control (Online supplement Table 1). Typical antipsychotics were used by 19 (32%) and atypical antipsychotics by 43 (72%) cases. The distribution of individual antipsychotic agents during lifetime use is described in more detail in Online supplement Table 2.

The cumulative lifetime antipsychotic doses and current antipsychotic doses of cases are reported in Table 2. Median lifetime antipsychotic dose-years as chlorpromazine equivalents were 29.2 dose-years for any antipsychotics, 9.6 for typical and 16.1 for atypical antipsychotics.

### 3.3 Cognitive functioning of cases and controls

The original cognitive test scores of cases and controls and results of the PCA are presented in Table 3. Cases performed significantly worse than controls on all cognitive tests and global cognition.

### 3.4 Correlations between antipsychotic dose-years, cognition and covariates

Higher cognitive composite score was significantly correlated with lower lifetime dose-years of any antipsychotics (p=0.004), higher age of illness onset (p=0.013), higher school marks at 16 years of age (p<0.001) and lower disorganisation symptoms (p<0.001) (Online supplement Table 3). Higher lifetime dose-years of any antipsychotics were significantly correlated with lower age of illness onset (p<0.001), higher number of lifetime hospital treatment days (p<0.001) and higher positive (p=0.002), negative (p=0.009) and disorganisation symptoms (p<0.001) (Online supplement Table 3). DAI total score did not correlate with cognition, lifetime antipsychotic dose-years of any antipsychotics, age of illness onset, lifetime hospital treatment days, PANSS symptoms or school marks at 16 years of age (Online supplement Table 3).

### 3.5 The association between lifetime cumulative antipsychotic dose-years and global cognitive functioning

**Fig. 1:**
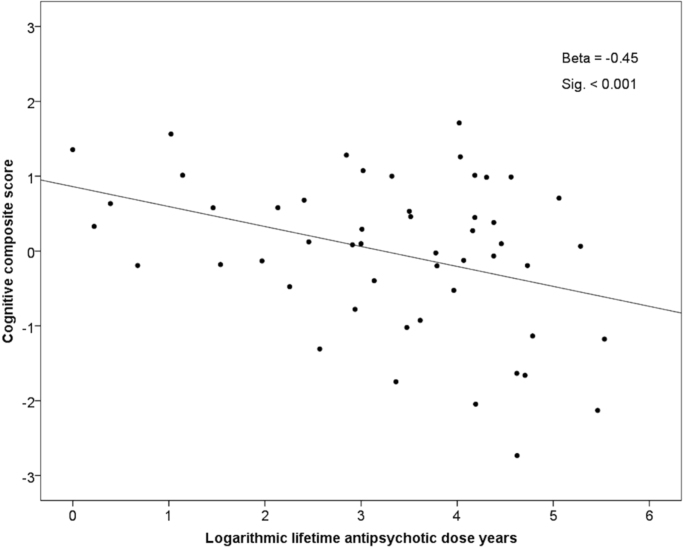
The association between lifetime dose-years of any antipsychotics and cognitive composite score at age 43 years in schizophrenia. *Higher lifetime antipsychotic dose-years associated with poorer cognitive composite scor*e. (Beta coefficient and statistical significance are from linear regression analysis with natural logarithm of dose-years of any antipsychotics as the predictor variable).

Higher lifetime cumulative dose-years of any antipsychotics were significantly associated with poorer cognitive composite score (p<0.001), when adjusted for gender and age of illness onset (p=0.005) (Table 4). This association remained significant, when adjusted (in addition to gender and age of illness onset) for remission (p=0.026), lifetime cumulative number of hospital treatment days (p=0.004), educational level (p=0.004), and current use of benzodiazepines (p=0.014) (Table 4). The association remained significant also, when gender, age of illness onset, remission, hospital treatment days and educational level were all included in the same regression model (p=0.016) (Table 4). However, the association between higher dose-years of any antipsychotics and poorer cognition did not remain significant, when school marks at 16 years of age were taken into account (Table 4). Fig. 1 demonstrates the unadjusted association between cumulative lifetime dose-years of any antipsychotics and cognitive composite score. Significant associations between higher antipsychotic dose-years and poorer cognition were found for both typical and atypical antipsychotics, when adjusted for gender and age of illness onset, educational level and for atypical antipsychotics also, when adjusted for lifetime hospital treatment days, and for typical antipsychotics, when adjusted for the current use of benzodiazepines (Table 4). For the full results, please see Table 4.

Regarding adjustment for symptoms, the association between higher dose-years of any antipsychotics and poorer cognition was non-significant, when adjusted for disorganisation symptoms (p=0.437). When adjusted for positive symptoms (p=0.020), negative symptoms (p=0.047) and gender, onset age, hospital treatment days, educational level and positive and negative symptoms in the same model (p=0.035) the association remained significant (Table 5).

Significant associations between antipsychotic dose-years and cognition remained in sensitivity analyses completed in the original data without imputation as well as in sensitivity analyses with inverse probability weighting to rule out the possible bias caused by the selective attrition of cases. In the original data without imputation the only exception was that the association between atypical antipsychotic dose-years and cognition did not remain significant, when adjusted for gender, age of illness onset and hospital treatment days. With inverse probability weighting there were additional significant results for higher typical antipsychotic dose-years and poorer cognition in models adjusted for gender, age of illness onset and remission (p=0.046) or hospital treatment days (p=0.031) or negative symptoms (p=0.034) and in the model adjusted for gender, age of illness onset, hospital treatment days, educational level and positive and negative symptoms (p=0.047). When cases with current benzodiazepine use (n=11), an intellectual disability diagnosis (n=6) or alcohol abuse diagnosis (n=6) were excluded from the analyses, the significant associations between any antipsychotics and cognition remained. Data of the sensitivity analyses are not shown but are available on request.

## 4 Discussion

In this study higher lifetime cumulative dose-years of any antipsychotics were significantly associated with poorer global cognition at the age of 43 years in schizophrenia, when the most important confounding factors related to duration and severity of illness were controlled for. However, the association did not remain when disorganisation symptoms and school marks at the age of 16 were taken into account. The cognitive effects of typical and atypical antipsychotics were similar.

### 4.1 Comparison with previous studies

Nearly all evidence of the cognitive effects of antipsychotics rests on relatively short-term clinical trials which have found mild to moderate cognitive improvements associated with both atypical (Désaméricq et al., 2014, Woodward et al., 2005) and typical (Mishara and Goldberg, 2004) antipsychotics in schizophrenia. However, a practice effect has been suggested to confound these findings of improvement and antipsychotics may only have minimal effects on cognition in the short-term (Goldberg et al., 2010, Szöke et al., 2008).

The association between antipsychotic dose and cognition has not been adequately analysed in the previous studies. Most studies included in meta-analyses do not report antipsychotic doses (Irani et al., 2011, Mesholam-Gately et al., 2009, Mishara and Goldberg, 2004). One meta-analysis of older people with schizophrenia (Irani et al., 2011) and another meta-analysis of typical antipsychotics and cognition (Mishara and Goldberg, 2004) did not find a significant association between antipsychotic dose and cognition. However, one meta-analysis found an association between higher antipsychotic dose and poorer processing speed (Knowles et al., 2010).

Our group found an association between higher cumulative antipsychotic exposure and brain volume loss (Veijola et al., 2014) and decline in verbal learning and memory (Husa et al., 2014) in the NFBC 1966 population (partially overlapping sample with the sample of this article) between ages 34 and 43 years. Now we extend our earlier findings by showing a significant association between higher cumulative antipsychotic dose and poorer global cognition at age 43 years in a larger sample.

The discrepancy between some data from previous meta-analyses and our present and earlier findings may be explained by differences in the length of follow-up (up to 2–3 years vs. 16.5 years in our study), setting (controlled vs. naturalistic), population (clinical vs. population-based), antipsychotic dose variable (cross-sectional vs. longitudinal, cumulative dose) as well as lack of reported medication dosage data that limits the power to find associations.

### 4.2 Interpretation of the results

Based on beta coefficients (-0.26 to −0.44) the effect sizes of the significant associations between higher lifetime antipsychotic dose and poorer cognition in this study are moderate.

The association between antipsychotic dose-years and cognition remained significant when adjusting for positive and negative symptoms. However, it did not remain significant when adjusting for disorganisation symptoms or school marks at 16 years of age. Disorganisation symptoms, such as stereotyped thinking, poor attention, disorientation, conceptual disorganisation and difficulty in abstraction (van der Gaag et al., 2006), in fact are clinical manifestations of cognitive deficits and inversely correlate with cognition (Minor and Lysaker, 2014). Premorbid school performance is associated with cognitive functioning at first-episode (Fuller et al., 2002) and poorer premorbid school performance predicts cognitive decline in midlife schizophrenia (Rannikko et al., 2015). In our data both disorganisation symptoms and school marks had significant and strong correlations with cognition. Thus, it may that the reduction of significant association in this case therefore reflects more overadjustment (Schisterman et al., 2009) rather than a genuine lack of association between higher lifetime antipsychotic dose and poorer cognition. Disorganisation symptoms had significant correlation with higher lifetime antipsychotics dose, whereas school marks at the age of 16 years did not. It is also possible that a part of the variance in the association between antipsychotic dose-years and cognition is explained by disorganisation symptoms and to some extent by premorbid school performance.

This study did not find differences in the cognitive effects of typical and atypical antipsychotics. The finding of similar effect may reflect no great differences between these groups or that due to small sample sizes there is not enough statistical power to detect possible differences. It is important to notice, that because most cases (85%) had used at least 3 different antipsychotic agents during their lives, it is impossible to separate between the effects of individual antipsychotic drugs on cognition in this sample.

### 4.3 Theoretical discussion

Evidence of the benefits of antipsychotic treatment is persuasive only during the first years of illness (Leucht et al., 2012). There is cumulating evidence that long-term and higher dose antipsychotic treatment may have potentially harmful effects on brain structure (Andreasen et al., 2013, Fusar-Poli et al., 2013, Veijola et al., 2014) and functioning (Abbott et al., 2013) as well as cognition (Husa et al., 2014, Knowles et al., 2010). Cognitive deficits in schizophrenia are associated with hypoactive prefrontal cortex and D2 receptor antagonist antipsychotics may additionally decrease the hypodopaminergic state of mesocortical pathways projecting to prefrontal cortex and worsen negative symptoms and cognitive impairments (Liemburg et al., 2012). Moreover, high-dose antipsychotic exposure resulting in D2 occupancy above 80% has been associated with neurocognitive impairment (Sakurai et al., 2013). In animal models, the adverse effects of antipsychotics on cognitive functioning have not occurred immediately but only after three to six months and these effects may be mediated by alterations in cholinergic function in the brain (Terry and Mahadik, 2007). In addition, sedative antipsychotic effects conveyed by histamine-1, muscarine and alfa-1-adrenergic receptors and 5-HT-2A antagonistic effects that inhibit glutaminergic function and may disturb mesolimbic and mesocortical functions have been hypothesised to explain negative cognitive effects of antipsychotics (Stahl, 2008). Dose reduction or discontinuation of antipsychotic treatment is associated with better functional remission rates after 7 years (Wunderink et al., 2013) and not using antipsychotics with fewer psychotic symptoms and less hospital treatment in 20 years of naturalistic follow-up in schizophrenia (Harrow et al., 2014). In the NFBC 1966 sample, being antipsychotic-free was associated with better outcome at age 34 years (Moilanen et al., 2013). Our results add to the above mentioned studies, supporting the potentially harmful effects of, in particular, high doses of antipsychotics in the long-term.

### 4.4 Strengths and limitations

A fundamental strength is the naturalistic, population-based study sample with extensive, prospectively collected information. Owing to the observational setting it was possible to investigate long-term and adverse effects of medication which cannot be detected in short-term clinical trials (Wang et al., 2011). The longitudinal data on lifetime cumulative antipsychotic exposure is unique. Because we had access also to medical records we were able to take into account known periods when patients were not taking medications when estimating lifetime antipsychotic doses, not only prescribed or purchased medication as most register or interview studies. Additionally, we measured DAI-10 which has been shown to predict antipsychotic adherence in some studies (Brain et al., 2013, Yang et al., 2012). Based on the DAI total score (Table 1) the participants had good adherence to antipsychotic treatment. Also finding no correlation between the DAI score at 43 years of age and lifetime antipsychotic dose or cognition (Online supplement Table 3) may further support adherence not being a significant confounder of the association between higher antipsychotic dose and poorer cognition. It should be noted, though, that we did not have any measure of adherence during the whole illness course.

A limitation is the relatively small sample size which decreases statistical power and likelihood to detect even moderate signals, especially in smaller subsamples (for example analysing the effects of typical and atypical antipsychotics). A non-standard neuropsychological test battery is also a potential limitation, though valid tests which measure the most essential cognitive dimensions similarly to standardised batteries (MATRICS, CANTAB, WAIS-III) were used. Because we did not study cognitive change, we cannot find out if the association between higher antipsychotic dose and poorer cognition is temporal or causal.

The selective attrition rising from the participating cases having markers of more severe illness and poorer functioning (lower age of illness onset and educational level, more often a narrow schizophrenia diagnosis and disability pension) compared with non-participants may introduce a bias in interpreting the results. However, in our population based sample the participating cases also had characteristics related to less severe illness and better functioning, e.g. 92% were outpatients, 43% even without psychiatric treatment contact and 28% were in remission. This indicates that there were both poorly and well-functioning cases in our sample. Moreover, based on the weighted sensitivity analyses, the selective attrition did not significantly affect the results of this study.

Even though the naturalistic design has its limitations e.g. in finding causal associations, RCTs are not possible or realistic in long follow-ups and naturalistic studies are the only option to get new information. Additionally, our epidemiologically sound, population based sample of mostly outpatients and some people without psychiatric treatment contact may reflect less severe illness than in many clinical settings and make the results less generalisable to some severe clinical populations but more generalisable to all individuals with schizophrenia.

To decrease the risk of residual confounding in our naturalistic setting with a long follow-up, the most important confounders related to duration and severity of illness were taken into account. However, individuals with more severe illness and poorer course of cognition may receive higher doses of antipsychotic medication, making higher doses a marker of a more serious illness course rather than cause of cognitive decline. Nonetheless it cannot be ruled out, especially with the cumulating evidence from other studies of potentially harmful effects of years of antipsychotic use on cognition, brain structures and functioning, that the findings reflect a true causal association.

### 4.5 Conclusions

To our knowledge, this is the first report of an association between cumulative lifetime antipsychotic dose and global cognition in midlife schizophrenia. Based on these data higher cumulative lifetime dose-years of antipsychotics may be associated with poorer cognitive performance at the age of 43 years. It is possible that large doses of antipsychotics influence the natural course of schizophrenia in midlife, for example by preventing or attenuating cognitive recovery.

## Conflict of interest

J.H. Barnett is an employee of and shareholder in Cambridge Cognition, a cognitive assessment company. P.B. Jones has been a member of Roche and Otsuka Scientific Advisory Boards 2012–2014. All other authors declare that they have no conflicts of interest.

## Role of the funding source

Funding for this study was provided by the Academy of Finland Grants 132 071, 278 286 and 268 336, the Sigrid Jusélius Foundation, the Brain & Behavior Research Foundation, the Jalmari and Rauha Ahokas Foundation, the Emil Aaltonen Foundation, the Finnish Cultural Foundation Lapland Regional Fund, the Northern Finland Health Care Support Foundation and the UK Medical Research Council Grant G0701911. J.H.B. was an employee of Cambridge Cognition. The funding bodies had no further role in study design; in the collection, management, analysis and interpretation of data; in the preparation, review or approval of the manuscript; or in the decision to submit the paper for publication.

## Figures and Tables

**Table 1:** Characteristics of the sample.

	Schizophrenia	Controls
***Sociodemographic factors***		
**Sex, n (%)**		
Males	33 (55%)	94 (49%)

**Education, n (%)**^a^		
Low	33 (56%)	71 (37%)
Middle	15 (25%)	46 (24%)
High	11 (19%)	73 (38%)

**Occupational status, n (%)**		
Working	18 (30%)	182 (95%)
**School marks at 16 years, mean (SD)**^a^	7.5 (0.9)	7.7 (0.9)

***Clinical factors***		
**Alcohol abuse dg, n (%)**^a^		
Yes	6 (10%)	9 (5%)
**Current use of alcohol (g/day), median (IQR)**^a^	1.2 (0–14.0)	5.7 (2.0–14.4)
**SOFAS, mean (SD)**^a^	50.7 (16.8)	84.6 (10.0)
**DAI, mean (SD)**^a^	5.6 (4.1)	
**Onset age (years), mean (SD)**	26.6 (6.3)	
**Number of hospital treatment days, median (IQR)**	210 (84–687)	

**Psychiatric treatment status, n (%)**		
No treatment contact	26 (43%)	
Non-regular outpatient treatment	7 (12%)	
Regular outpatient treatment	22 (37%)	
Inpatient/institution	5 (8%)	

**Diagnosis, n (%)**		
Schizophrenia	50 (83%)	
Schizophreniform disorder	2 (3%)	
Schizoaffective disorder	6 (10%)	
Delusional disorder	2 (3%)	

***Symptom severity***		
**CGI, mean (SD)**	4.5 (1.4)	
**PANSS, mean (SD)**^a^	66.6 (23.5)	

**Remission, n (%)**^a^		
Yes	16 (28%)	

SOFAS=Social and Occupational Functioning Assessment Scale, CGI=Clinical Global Impression, DAI=Drug Attitude Inventory, PANSS=Positive and Negative Syndrome Scale, IQR=interquartile range. Psychiatric treatment status: non-regular outpatient treatment=contact less than once per month or of unknown frequency, regular outpatient treatment=1–4 times per month and inpatient/institution=being in psychiatric hospital treatment or sheltered home.

a There were missing data for 1 case and 1 control in education, 1 control and 2 cases in school marks at 16 years, 1 control in alcohol abuse dg, 1 case and 1 control in current use of alcohol, 2 controls in SOFAS, 7 cases in DAI, 2 cases in PANSS, 2 cases in remission.

**Table 2:** Lifetime antipsychotic dose-years^a^ and current antipsychotic dose^b^ in schizophrenia (n=60).

	Lifetime antipsychotic dose-years Md (IQR)	Current antipsychotic dose Md (IQR)
Any antipsychotics	29.2 (12.7–69.6)	225 (106.3–500.0)
Typical antipsychotics	9.6 (0.8–32.7)	0 (0–57.5)
Atypical antipsychotics	16.1 (2.6–37.9)	200.0 (0–475.0)

Md=median, IQR=interquartile range.

a Kroken et al. (2009).

b Chlorpromazine equivalents.

**Table 3:** Original values of the cognitive tests and the cognitive composite score based on principal component analysis in schizophrenia (n=60) and controls (n=191).

	Schizophrenia mean (SD)	Controls mean (SD)	Sig^a^
AIM, Total score	41.5 (8.0)	48.3 (5.0)	<0.001
CVLT, Immediate free recall of trials 1–5	43.7 (15.4)	55.2 (9.0)	<0.001
VOLT, Total score	60.1 (9.8)	67.7 (5.4)	<0.001
Verbal fluency, Total score	47.5 (12.9)	58.4 (12.3)	<0.001
Visual series (WMS-III), Total score	15.0 (4.1)	17.8 (2.8)	<0.001
Vocabulary (WAIS-III), Total score	34.1 (14.7)	45.3 (11.4)	<0.001
Digit span (WAIS-III), Total score	14.1 (3.9)	16.4 (3.9)	<0.001
Matrix reasoning (WAIS-III), Total score	14.4 (5.8)	19.5 (3.6)	<0.001
Cognitive composite score^b^	−0.98 (1.2)	0.29 (0.7)	<0.001

AIM=Abstraction Inhibition and Working Memory task, CVLT=California Verbal Learning Test, VOLT=Visual Object Learning Test, WAIS-III=Wechsler Adult Intelligence Scale – Third Edition, WMS-III - Wechsler Memory Scale – Third Edition.

a Difference between cases and controls.

b Principal Component Analysis. Rotation method: Promax with Kaiser Normalisation.

**Table 4:** The association between lifetime antipsychotic dose-years and cognitive composite score at 43 years of age in schizophrenia in linear regression analysis.

Covariates in the model	Any antipsychotics			Typical antipsychotics			Atypical antipsychotics		
	B (SE)	Beta	Sig	B (SE)	Beta	Sig	B (SE)	Beta	Sig
Unadjusted	**−0.33 (0.09)**	**−0.44**	**<0.001**	**−0.27 (0.08)**	**−0.43**	**<0.001**	**−0.20 (0.08)**	**−0.31**	**0.015**
Gender, onset age^a^	**−0.27 (0.10)**	**−0.36**	**0.005**	**−0.23 (0.11)**	**−0.37**	**0.036**	**−0.17 (0.08)**	**−0.26**	**0.028**
Gender, onset age, remission	**−0.25 (0.11)**	**−0.33**	**0.026**	−0.19 (0.12)	−0.31	0.102	−0.15 (0.09)	−0.23	0.085
Gender, onset age, hospital treatment days^b^	**−0.33 (0.11)**	**−0.43**	**0.004**	−0.23 (0.12)	−0.36	0.050	**−0.19 (0.09)**	**−0.29**	**0.036**
Gender, onset age, educational level	**−0.28 (0.10)**	**−0.37**	**0.004**	**−0.24 (0.11)**	**−0.37**	**0.035**	**−0.18 (0.08)**	**−0.27**	**0.025**
Gender, onset age, school marks at 16 years^c^	−0.15 (0.09)	−0.20	0.099	−0.09 (0.11)	−0.14	0.407	−0.12 (0.07)	−0.19	0.081
Gender, onset age, current use of benzodiazepines	**−0.24 (0.10)**	**−0.32**	**0.014**	**−0.22 (0.11)**	**−0.34**	**0.049**	−0.14 (0.08)	−0.22	0.086
Gender, onset age, remission, hospital treatment days, educational level	**−0.31 (0.13)**	**−0.41**	**0.016**	−0.31 (0.19)	−0.31	0.111	−0.27 (0.15)	−0.27	0.072
Gender, onset age, remission, hospital treatment days, school marks at 16 years^b^	−0.14 (0.12)	−0.18	0.262	−0.08 (0.18)	−0.08	0.660	−0.18 (0.13)	−0.18	0.172

B=unstandardised regression coefficient, SE=standard Error, Beta=standardised regression coefficient, Sig=statistical significance. Statistically significant results in **bold**.

a The adjusted R^2^ of the model without antipsychotic dose-years was 0.135. When antipsychotic dose-years were included in the model, the corresponding R^2^s were 0.250 for any, 0.188 for typical and 0.203 for atypical antipsychotics.

b Lifetime cumulative number of hospital treatment days (logarithmic transformation).

c Mean of school marks at 16 years of age.

**Table 5:** The association between lifetime antipsychotic dose-years and cognitive composite score at 43 years of age in schizophrenia in linear regression analysis, models adjusted with PANSS.^a^

Covariates in the model	Any antipsychotics			Typical antipsychotics			Atypical antipsychotics		
	B (SE)	Beta	Sig	B (SE)	Beta	Sig	B (SE)	Beta	Sig
Gender, onset age, positive symptoms	**−0.25 (0.11)**	**−0.32**	**0.020**	−0.20 (0.12)	−0.31	0.098	−0.15 (0.09)	−0.23	0.087
Gender, onset age, negative symptoms	**−0.20 (0.10)**	**−0.27**	**0.047**	−0.19 (0.11)	−0.30	0.084	−0.11 (0.09)	−0.16	0.213
Gender, onset age, disorganisation symptoms	−0.08 (0.10)	−0.10	0.437	−0.08 (0.10)	−0.13	0.445	−0.02 (0.08)	−0.03	0.829
Gender, onset age, hospital treatment days, educational level, positive symptoms, negative symptoms	**−0.26 (0.12)**	**−0.34**	**0.035**	−0.32 (0.19)	−0.32	0.083	−0.19 (0.15)	−0.19	0.227

B=unstandardised regression coefficient, SE=Standard Error, Beta=standardised regression coefficient, Sig=statistical significance. Statistically significant results in **bold**.

a The PANSS (Positive and Negative Syndrome Scale; Kay et al., 2000) was based on a PANSS specific interview and divided into positive, negative and disorganisation symptoms based on the factor structure described by van der Gaag et al. (2006).
