# Supplementary material for: Lifetime antipsychotic medication and cognitive performance in schizophrenia at age 43 years in a general population birth cohort
Source: Psychiatry Res. 2017 Jan;247:130–8. doi: 10.1016/j.psychres.2016.10.085 (PMC5241225; doi:10.1016/j.psychres.2016.10.085)
Supplement: Supplementary file 1 — Supplementary material [file mmc1.doc]

**Online supplement Table 1.** Current use of psychiatric medication at the age of 43 years among cases and controls.

|  | **Schizophrenia**  Last 3 months  n (%) | **Controls**  Last 3 months  n (%) |
| --- | --- | --- |
| **Use of any psychiatric medication** |  |  |
| yes | 53 (88%) | 16 (8%) |
| **Typical antipsychotics** | 19 (32%) | - |
| **Atypical antipsychotics** | 43 (72%) | 1 (0.5%) |
| **Both typical and atypical antipsychotics** | 11 (18%) | - |
| **Antidepressants** | 13 (22%) | 13 (7%) |
| **Benzodiazepines** | 11 (18%) | 3 (2%) |
| **Other psychiatric medication** | 12 (20%) | 2 (1%) |

Benzodiazepines included chlordiazepoxide, clonazepam, diazepam, lorazepam, oxazepam and temazepam. Other psychiatric medication included carbamazepine, lamotrigine, sodium valproate, hydroxyzine, melatonin and zopiclone. None of the cases was using anticholinergic medication. One control with antipsychotic medication was using 25 mg of quetiapine daily as a sleeping aid.

**Online supplement Table 2.** Lifetime use of antipsychotic medication in schizophrenia cases (n=60).

| **Name of the medication** | **Number of schizophrenia cases who have used the medication** |
| --- | --- |
| **Typical antipsychotics** |  |
| chlorpromazine | 22 (37%) |
| chlorprothixene | 15 (25%) |
| dixyrazine | 1 (2%) |
| flupentixol | 4 (7%) |
| fluphenazine | 4 (7%) |
| haloperidol | 40 (67%) |
| levomepromazine | 26 (43%) |
| melperone | 3 (5%) |
| molindone | 2 (3%) |
| periciazine | 1 (2%) |
| perphenazine | 36 (60%) |
| pimozide | 1 (2%) |
| pipotiazine | 3 (5%) |
| promazine | 20 (33%) |
| remoxipride | 2 (3%) |
| sulpiride | 7 (12%) |
| thioproperazine | 1 (2%) |
| thioridazine | 38 (63%) |
| zuclopenthixol | 23 (38%) |
| **Atypical antipsychotics** |  |
| aripiprazole | 11 (18%) |
| asenapine | 1 (2%) |
| clozapine | 16 (27%) |
| olanzapine | 31 (52%) |
| quetiapine | 19 (32%) |
| risperidone | 36 (60%) |
| sertindole | 3 (5%) |
| ziprasidone | 1 (2%) |
| **Number of medications used per person** |  |
| 0 | 1 (2%) |
| 1-2 | 9 (15%) |
| 3-5 | 20 (33%) |
| 6-9 | 18 (30%) |
| 10-^1^ | 12 (20%) |

^1^Maximally two persons had used up to 14 different antipsychotic agents.

**Online supplement Table 3.** Correlations between the cognitive composite score, lifetime dose-years of any antipsychotics and covariates.

|  | Cognitive composite score | | Lifetime dose-years of any antipsychotics | | DAI Total score | |
| --- | --- | --- | --- | --- | --- | --- |
|  | Spearman’s  correlation | Sig. | Spearman’s  correlation | Sig. | Spearman’s  correlation | Sig. |
|  |  |  |  |  |  |  |
| Lifetime dose-years of any antipsychotics | **-0.383** | **0.004** |  |  |  |  |
| DAI Total score | -0.118 | 0.430 | 0.215 | 0.122 |  |  |
| Onset age | **0.337** | **0.013** | **-0.544** | **<0.001** | -0.014 | 0.923 |
| Lifetime hospital treatment days | -0.231 | 0.092 | **0.664** | **<0.001** | 0.095 | 0.498 |
| PANSS positive symptoms | -0.056 | 0.695 | **0.396** | **0.002** | 0.068 | 0.637 |
| PANSS negative symptoms | -0.270 | 0.053 | **0.340** | **0.009** | 0.061 | 0.672 |
| PANSS disorganisation symptoms | **-0.584** | **<0.001** | **0.550** | **<0.001** | 0.056 | 0.698 |
| School marks at 16 years^1^ | **0.604** | **<0.001** | -0.176 | 0.186 | -0.217 | 0.127 |

DAI = Drug Attitude Inventory, PANSS = Positive and Negative Syndrome Scale. ^1^Mean of school marks in all subjects at 16 years of age. Sample size varies from 51 to 60. Significant results in **bold**.
